# Treatment of Cerebral Cavernous Malformations Presenting With Seizures: A Systematic Review and Meta-Analysis

**DOI:** 10.3389/fneur.2020.590589

**Published:** 2020-10-26

**Authors:** Xiangyu Gao, Kangyi Yue, Jidong Sun, Yuan Cao, Boyan Zhao, Haofuzi Zhang, Shuhui Dai, Lei Zhang, Peng Luo, Xiaofan Jiang

**Affiliations:** Department of Neurosurgery, Xijing Hospital, Fourth Military Medical University, Xi'an, China

**Keywords:** brain cavernous hemangioma, seizure, neurosurgery, radiosurgery, meta-analysis

## Abstract

**Background:** Cerebral cavernous malformations (CCMs) presenting with seizures can be treated with neurosurgery or radiosurgery, but the ideal treatment remains unclear. Currently, there is no adequate randomized controlled trial comparing surgical treatment and radiotherapy for epileptogenic CCMs. Therefore, we conducted a systematic review and meta-analysis of available data from published literature to compare the efficacy and safety of neurosurgery and radiosurgery for epileptogenic CCMs.

**Methods:** We performed a comprehensive search of the Ovid MEDLINE, Web of Science, PubMed, China Biological Medicine and China National Knowledge Infrastructure databases for studies published between January 1994 and October 2019. The search terms were as follows: “epilepsy,” “seizures,” “brain cavernous hemangioma,” “cerebral cavernous malformation,” “cerebral cavernous hemangioma,” “hemangioma, cavernous, central nervous system.” Two researchers independently extracted the data and reviewed all the articles. We compared the advantages and disadvantages of the two treatments.

**Results:** A total of 45 studies were included in our analysis. Overall, the seizure control rate was 79% (95% CI: 75–83%) for neurosurgery and 49% (95% CI: 38–59%) for radiosurgery. In the neurosurgery studies, 4.4% of patients experienced permanent morbidity, while no patients in the radiotherapy studies had permanent morbidity. In addition, the results of subgroup analysis showed that ethnicity, CCMs location and average lesion number are likely significant factors influencing the seizure outcome following treatment.

**Conclusions:** The epilepsy control rate after neurosurgery was higher than that after radiosurgery, but neurosurgery also had a relatively higher rate of permanent morbidity.

## Introduction

Cerebral cavernous malformations (CCMs), also known as cavernous angiomas, have an incidence of 0.1–0.5% and account for 5–10% of cerebral and spinal vascular malformations (1–3). CCMs are benign vascular lesions that can occur anywhere in the brain parenchyma or leptomeninges but mainly occur in the supratentorial region. They are abnormal low-flow blood vessels in the brain consisting of expanded, thin-walled capillary clusters filled with hemosiderin deposits. CCMs can manifest as central nervous system bleeding and other neurological defects based on their location, and 40–70% of supratentorial cavernous malformations tend to have seizures as the first symptom (2–4). A total of 35–40% of CCM patients develop medically refractory epilepsy. The vascular morphology of CCMs is fragile and prone to repeated microbleeds, leading to reactive gliosis and hemosiderin deposition in adjacent brain tissues (2, 5, 6). Thus, the resulting ischemia, venous hypertension, glial hyperplasia, and inflammatory responses can all induce seizures and involve the brain parenchyma near these lesions. Of all cerebral vascular malformations, CCMs are the most common epileptic substrate. Seizures are the most common symptoms of supratentorial CCMs (7, 8). Epilepsy is known to significantly reduce quality of life and cause severe morbidity, and antiepileptic drugs (AEDs) often have undesirable side effects (9–11). Therefore, eliminating epilepsy is an important and often underestimated therapeutic goal in managing these lesions.

However, the ideal treatment remains unclear. Microsurgery is considered the standard treatment for intractable epilepsy caused by CCMs. Surgical removal can prevent seizures in 50–90% of patients (12). In the past few decades, with the application of advanced technology such as diffusion tensor imaging (DTI) and electrophysiological monitoring, surgical intervention had produced better results (13). Additionally, recent studies had confirmed that microsurgery could exhibit great seizure control rate (14, 15). However, the risk of surgical morbidity and mortality is high when the lesion is located in deep or eloquent areas (16–19). Stereotactic radiosurgery is another option for the treatment of CCMs, especially in high-risk patients (20, 21). In the treatment of epileptogenic CCMs, several authors have indicated that gamma knife radiosurgery (GKRS) can provide good seizure control (22–24). Currently, there is no adequate randomized controlled trial comparing surgical treatment and radiotherapy for epileptogenic CCMs. Therefore, we conducted a systematic review and meta-analysis of available data from published literature to compare the efficacy and safety of neurosurgery and radiosurgery for epileptogenic CCMs.

### Methods

The present study was performed according to the Preferred Reporting Items for Systematic Reviews and Meta-Analyses guidelines (PRISMA) (25).

### Search Strategy

**Figure 1:**
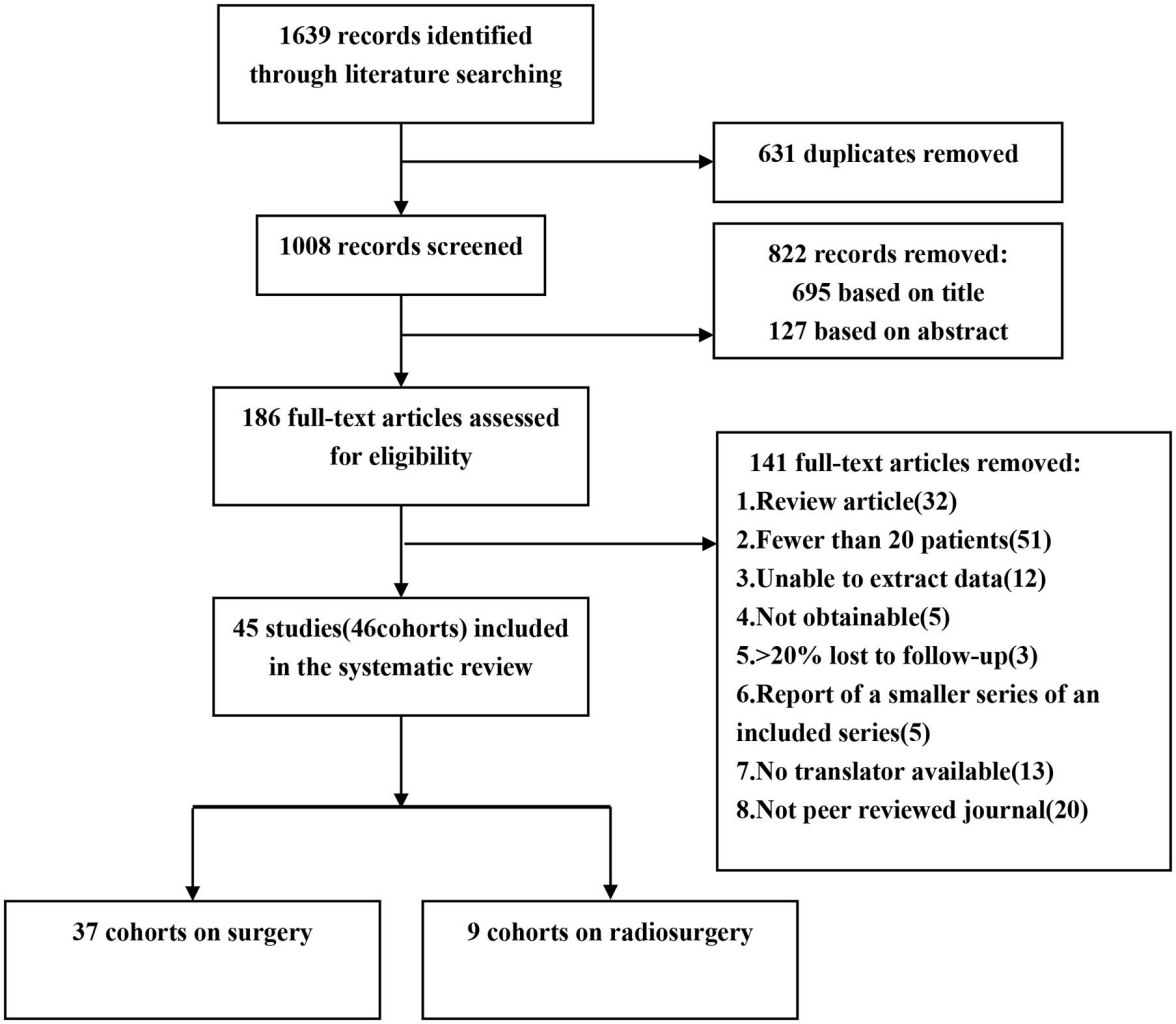
Flow chart of the data search.

We performed a comprehensive search of the Ovid MEDLINE, Web of Science, PubMed, China Biological Medicine and China National Knowledge Infrastructure databases for studies published between January 1994 and October 2019. The search terms were as follows: “epilepsy,” “seizures,” “brain cavernous hemangioma,” “cerebral cavernous malformation,” “cerebral cavernous haemangioma,” “hemangioma, cavernous, central nervous system.” We retrieved the original articles of cohort studies published in peer-reviewed journals. We included eligible studies published in Chinese and English, while studies in other languages were excluded because we did not have translators (Figure 1).

### Assessment of Eligibility

Two independent reviewers selected eligible studies based on the Patient, Intervention, Comparison, Outcome, and Study design (PICOS) guidelines (23): (1) Participants: patients' CCMs had to be confirmed by MRI or pathological examination; (2) Interventions: neurosurgery or radiosurgery; (3) Comparison: not applicable; (4) Outcome: seizure outcome estimated by Engel's classification; (5) Study designs: retrospective cohort study; the sample sizes of the studies had to be >20; studies must have described the follow-up time, and the follow-up rate had to be >80%. If the institution or author published multiple studies using the same cohort, only the report with the largest sample size was included for analysis. Case reports, reviews, meta-analyses, letters and conference articles were excluded.

### Risk of Bias Assessment

The Newcastle-Ottawa Scale (NOS) was used to assess the quality of the included studies. The NOS score is used to assess three major components: selection, comparability, and exposure. Studies are defined as high quality when scoring ≥5. Two reviewers independently evaluated the quality of the studies and resolved disagreements by discussion.

### Data Extraction

A total of 1,639 articles were retrieved in our initial search. Two researchers (Xiangyu Gao and Peng Luo) independently extracted the data and reviewed all the articles. First, two researchers screened the titles and abstracts of the retrieved literature. They then evaluated the full-texts of relevant articles to determine their eligibility. Opinion was sought from a senior investigator (Xiaofan Jiang) if the two researchers could not reach an agreement. Finally, 45 of 1,639 articles met the inclusion criteria. Two investigators extracted the following data from each eligible study: first author's last name, publication date, year of patients, total number of patients, number of female patients, mean follow-up time, mean age, mean duration of epilepsy, lesion location, post-operative seizure outcome, mortality, temporary morbidity and permanent morbidity (6, 24, 26–68) (Table 1). The term “mortality” is defined as patients' death attributed to CCMs or treatment. Temporary morbidity includes transient brain edema after surgery, new or worse neurological deficits, and a range of other complications, all of which can eventually be fully cured. Permanent morbidity includes memory deficits and persistent focal neurological deficits.

**Table 1:** Basic patient characteristics of each included cohort.

**First author,** **year of publication**	**Year**	**Number of treated patients**	**Number of female patients (%)**	**Mean age (years)**	**Mean duration of seizure (years)**	**Mean duration of follow-up (years)**	**Engel class I (%)**	**Engel class II-IV (%)**	**Mortality(%)**	**Temporary morbidity(%)**	**Permanent morbidity(%)**
**Neurosurgery (*****n*** **=** **37)**											
Cohen, 1995	1981–1992	51	29 (56.9)	34.9	4.7	5.0	NA	NA	1.0	NA	NA
Casazza, 1996	1988–1992	47	18 (38.3)	32.4	5.3	4.0	NA	NA	NA	17.0	NA
Zevgaridi, 1996	1984–1993	77	41 (53.2)	32.3	4.8	2.5	NA	NA	0.0	36.4	2.6
Cappabiar, 1997	1985–1994	35	21 (60.0)	28.8	NA	NA	NA	NA	NA	NA	NA
Baumann, 2006	NA	27	NA	36.3	12.0	3.0	8 (29.6)	19 (70.4)	0.0	NA	7.4
D'Angelo, 2006	1992–2005	69	NA	NA	NA	NA	57 (82.6)	12 (17.4)	0.0	27.5	NA
Ferroli, 2006	1988–2003	163	NA	33.4	4.5	NA	NA	NA	0.0	13.5	NA
Hamen, 2007	NA	30	13 (43.3)	39.4	10.8	NA	17 (56.7)	12 (40.0)	NA	NA	NA
Huo, 2008	2003–2006	58	NA	NA	NA	1.8	42 (72.4)	16 (17.6)	0.0	7.0	0.0
Stavrou, 2008	1981–2004	53	22 (41.5)	NA	3.6	8.1	45 (84.9)	8 (15.1)	0.0	9.4	17.0
Wang, 2008	1998–2005	25	10 (40.0)	39.0	NA	NA	22 (88.0)	3 (12.0)	0.0	8.0	NA
Chang, 2009	1996–2006	44	NA	NA	NA	NA	32 (72.7)	12 (27.3)	NA	NA	NA
Yeon, 2009	1995–2005	60	23 (38.3)	NA	NA	NA	50 (83.3)	10 (16.7)	0.0	1.7	15.0
Guo, 2010	2003–2008	57	18 (31.6)	27.4	2.6	1.8	45 (78.9)	10 (17.5)	0.0	NA	0.0
Chen, 2011	2003–2008	27	11 (40.7)	29.0	NA	3.2	16 (59.3)	11 (40.7)	0.0	11.1	0.0
Hugelshofer, 2011	1974–2004	36	NA	NA	NA	NA	26 (72.2)	10 (27.8)	NA	8.3	NA
Kivelev, 2011	1980–2009	39	29 (74.4)	NA	3.0	6.0	30 (76.9)	9 (23.1)	NA	30.0	NA
Gross, 2013	1997–2011	48	NA	NA	NA	NA	46 (95.8)	2 (4.2)	NA	6.3	8.3
Kwon, 2013	1995–2008	56	29 (51.8)	37.5	NA	7.3	46 (82.1)	10 (17.9)	NA	NA	NA
Sommer, 2013	2002–2012	26	14 (53.8)	39.1	NA	4.0	21 (80.8)	5 (19.2)	0.0	7.7	11.5
Von der Brelie, 2013	1988–2010	118	47 (40.2)	38.9	10.9	NA	NA	NA	6.8	17.8	0.0
Wang, 2013	2000–2008	132	64 (48.5)	39.3	2.3	NA	NA	NA	0.0	7.6	3.8
Jin, 2014	2011–2012	36	15 (41.7)	37.8	0.5	1.5	28 (77.8)	8 (22.2)	0.0	NA	0.0
Kim, 2014	1989–2008	46	23 (50.0)	31.2	3.6	8.0	NA	NA	0.0	2.2	0.0
Wang, 2014	2009–2013	30	12 (40.0)	34.6	2.3	1.3	25 (83.3)	5 (16.7)	0.0	NA	0.0
Ge, 2015	2005–2013	25	NA	NA	NA	NA	23 (92.0)	1 (4.0)	4.0	NA	NA
Meguins, 2015	2000–2012	21	8 (38.1)	34.4	12.0	3.1	13 (61.9)	8 (38.1)	0.0	9.5	14.3
Shan, 2015	2008–2012	52	21 (40.4)	26.8	NA	3.2	42 (80.8)	10 (19.2)	0.0	0.0	0.0
Sun, 2015	2008–2014	51	29 (56.9)	NA	NA	NA	40 (78.4)	11 (21.6)	0.0	11.8	0.0
Vale, 2015	1999–2011	34	18 (52.9)	37.0	3.8	5.5	29 (85.3)	5 (14.7)	0.0	3.0	0.0
Wu, 2015	2010–2014	27	9 (33.3)	9.4	0.7	3.1	25 (92.6)	2 (7.4)	0.0	7.4	0.0
Hou, 2016	2012–2015	56	26 (46.4)	26.8	NA	NA	43 (76.8)	13 (23.2)	0.0	28.6	0.0
Dammann, 2017	NA	41	18 (43.9)	28.0	0.2	5.8	NA	NA	NA	19.5	NA
He, 2017	2005–2009	181	81 (44.8)	33.4	3.0	6.9	145 (80.1)	36 (19.9)	0.0	5.0	0.0
Barzaghi, 2018	2010–2017	43	NA	NA	NA	NA	34 (79.1)	9 (20.9)	0.0	48.8	9.3
Yang, 2018	2004–2014	47	20 (42.6)	34.3	9.9	5.3	39 (83.0)	8 (17.0)	0.0	0.0	14.9
Lin, 2018	2004–2016	27	15 (55.6)	15.0	2.3	6.3	21 (77.8)	6 (22.2)	0.0	7.4	14.8
**GKRS (*****n*** **=** **9)**											
Regis, 2000	1991–1997	49	23 (46.9)	36.0	7.5	2.0	26 (53.1)	23 (46.9)	0.0	4.1	0.0
Wang, 2009	2002–2008	25	NA	NA	NA	NA	21 (84.0)	NA	NA	NA	0.0
Wang, 2010	1995–2005	44	NA	NA	NA	NA	24 (54.5)	20 (45.5)	NA	NA	0.0
Chen, 2011	1997–2005	30	11 (36.7)	35.0	NA	2.8	8 (26.7)	22 (73.3)	0.0	40	0.0
Jia, 2014	1996–2010	48	NA	NA	NA	3.1	23 (47.9)	25 (52.1)	0.0	NA	0.0
Kida, 2015	1991–2012	27	NA	NA	NA	NA	13 (48.1)	14 (51.9)	NA	NA	NA
He, 2016	2008–2013	36	16 (44.4)	30.0	NA	4.0	13 (36.1)	23 (63.9)	0.0	22.2	0.0
Xu,2017	2012–2016	24	NA	NA	NA	3.6	11 (45.8)	13 (54.2)	0.0	NA	0.0
Yang, 2019	2015–2017	60	28 (46.7)	41.9	4.8	3.0	24 (40.0)	36 (60.0)	0.0	13.3	0.0

*GKRS, gamma knife radiosurgery; NA, unknown*.

### Statistical Analysis

We pre-specified the following characteristics of the included cohorts as the baseline covariates of interest: mean duration of epilepsy, cohort midyear (defined as the middle of the year in which the treatment occurred), mean age of the patients, percentage of female patients, CCM location, percentage of patients who died, percentage of patients with temporary morbidity and percentage of patients with permanent morbidity. We used the Mann-Whitney *U*-test to evaluate the difference in the proportion of these characteristics between the neurosurgery and radiosurgery groups, with a *p*-value < 0.05 indicating a significant difference. The seizure outcome data were estimated by Engel's classification. Engel class I represented complete freedom from seizures since the operation, and Engel classes II-IV represented not seizure-free. To standardize the evaluation of the study results, we calculated the proportion of patients in Engel class I in each group. Meta-analysis software (version 14.2, Stata) was used to calculate the overall proportions. Statistical heterogeneity was evaluated by the *I*^2^ statistic. If *I*^2^ > 50%, we used a random effects model to analyze the assumption. Otherwise, we used a fixed effects model. Sensitivity analysis was performed to investigate the impact of an individual study on the overall risk assessment by omitting one study at a time. Publication bias was evaluated qualitatively examining the funnel plot and quantitatively by Egger's test, which was considered statistically asymmetrical when the *p*-value < 0.1.

## Results

### Systematic Literature Review

After screening, 45 studies (46 cohorts) involving 2,356 patients were identified. Thirty-seven studies described a total of 2013 patients who underwent neurosurgery, and nine studies described a total of 343 patients who underwent radiosurgery. Four (9%) cohorts examined patients from multiple centers, and the remaining 42 (91%) cohorts examined patients from a single center. Twenty-five (55%) cohorts were from Asia, 14 (30%) cohorts were from Europe, 5 (11%) cohorts were from North America, 1 (2%) cohort was from South America, and 1 (2%) cohort was from Oceania. All 45 studies were published between 1995 and 2019. Twenty-eight studies (62%) described the mean or median duration of follow-up. Thirty-seven studies (80%) described post-operative seizure outcomes. We found statistically significant differences in the CCM location and proportion of patients with permanent morbidity between the neurosurgery and radiosurgery groups. GKRS is more suitable for CCM lesions located in the parietal lobe and occipital lobe, while neurosurgery is more suitable for temporal lobe lesions. Compared with patients in the radiosurgery group, patients in the neurosurgery group had a higher incidence of permanent morbidity after surgery (Table 2).

**Table 2:** Characteristics of the included cohorts.

	**Overall (*****n*** **=** **46)**			**Neurosurgery (*****n*** **=** **37)**			**Radiosurgery (*****n*** **=** **9)**		
**Study characteristics**	**Cohorts (%)^**a**^**	**Patients**	**Median (range)**	**Cohorts (%)**	**Patients**	**Median (range)**	**Cohorts (%)**	**Patients**	**Median (range)**
Patients treated	46 (100)	2356	44 (21–181)	37 (100)	2013	46 (21–181)	9 (100)	343	36 (24–60)
Duration of epilepsy, y	23 (50)	1393	3.8 (0.2–12)	21 (57)	1284	3.6 (0.2–12)	2 (22)	109	6.2 (4.8–7.5)
Duration of follow–up, y	28 (61)	1307	3.4 (1.3–8.1)	22 (59)	1060	4 (1.3–8.1)	6 (67)	247	3.1 (2–4)
Midyear, y	43 (93)	2258	2004 (1987–2016)	34 (92)	1915	2005 (1987–2014)	9 (100)	343	2003 (1994–2016)
Age, y	30 (65)	1662	34.4 (9.4–41.9)	26 (70)	1487	33.9 (9.4–39.4)	4 (44)	175	35.5 (30.0–41.9)
Female, %	32 (70)	1675	44 (32–74)	28 (76)	1500	44 (32–74)	4 (44)	175	46 (37–47)
**CMs location**									
Frontal, %	31 (74)	1417	28 (0–100)	26 (70)	1180	28.5 (0–100)	5 (56)	237	25 (18–33)
Temporal,%	35 (76)	1817	47 (0–100)	30 (81)	1580	49 (0–100)^*^	5 (56)	237	28 (23–47)^*^
Parietal,%	30 (65)	1387	14 (0–39)	25 (68)	1150	10 (0–29)^**^	5 (56)	237	28 (10–39)^**^
Occipital,%	29 (63)	1341	5 (0–27)	24 (65)	1104	4 (0–25)^*^	5 (56)	237	7 (4–27)^*^
Others,%	29 (63)	1341	1.3 (0–26)	24 (65)	1104	2 (0–26)	5 (56)	237	0 (0–8)
Mortality,%	34 (74)	1884	0 (0–6.8)	28 (76)	1637	0 (0–7)	6 (67)	247	0 (0)
Temporary morbidity,%	31 (67)	1797	9 (0–49)	27 (73)	1622	8 (0–49)	4 (44)	175	18 (4–40)
Permanent morbidity,%	32 (70)	1668	0 (0–17)	24 (65)	1352	0 (0–17)^*^	8 (89)	316	0 (0)^*^

a The percentage is the number of cohorts reporting a particular study characteristic divided by the total number of cohorts.

* P < 0.05 and

** P < 0.01, showing a significant difference in the median ratio between the group describing neurosurgery and the group describing radiosurgery.

*CMs, cavernous malformations*.

### Seizure Outcomes

**Figure 2:**
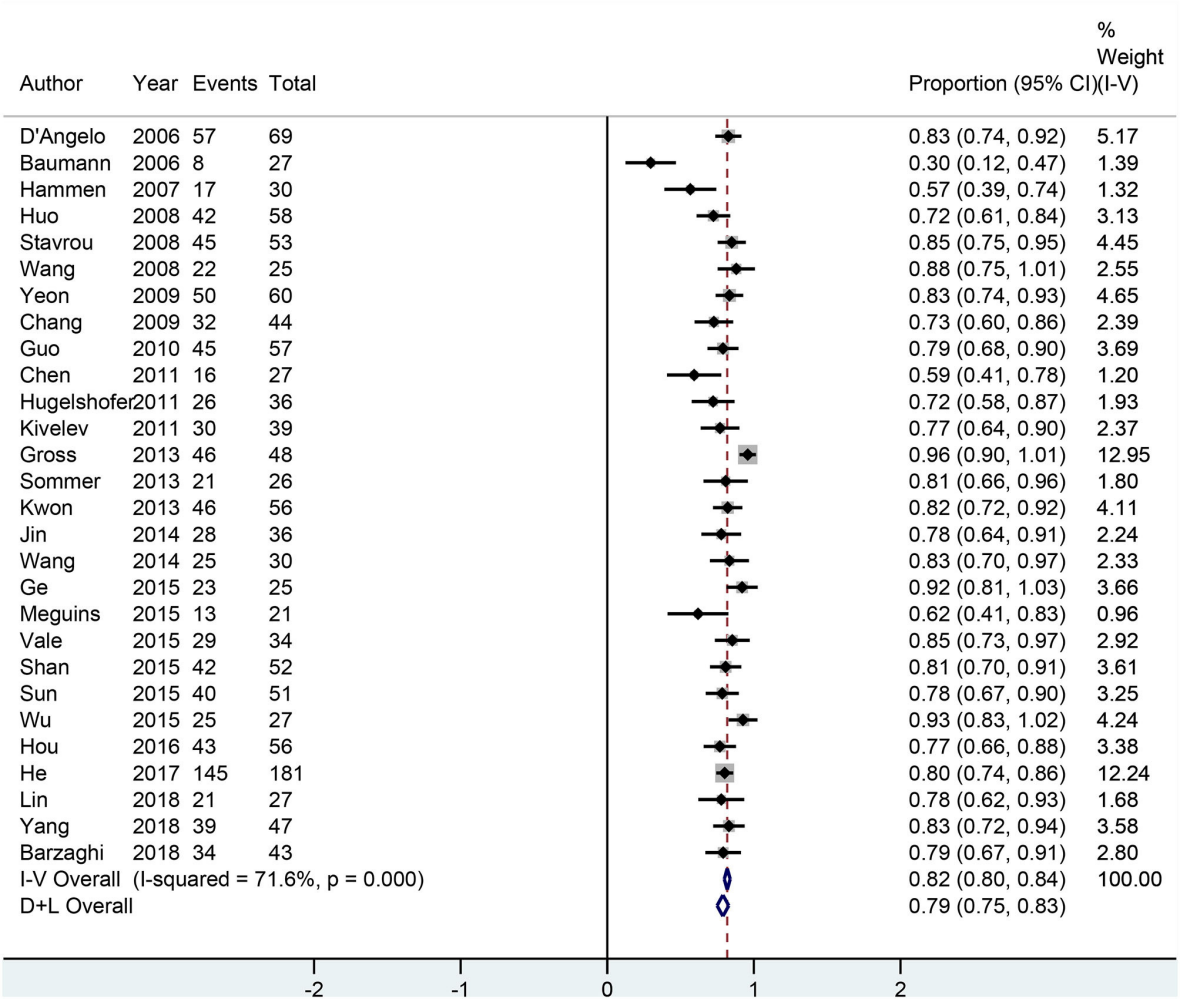
Forest plot of neurosurgery studies.

**Figure 3:**
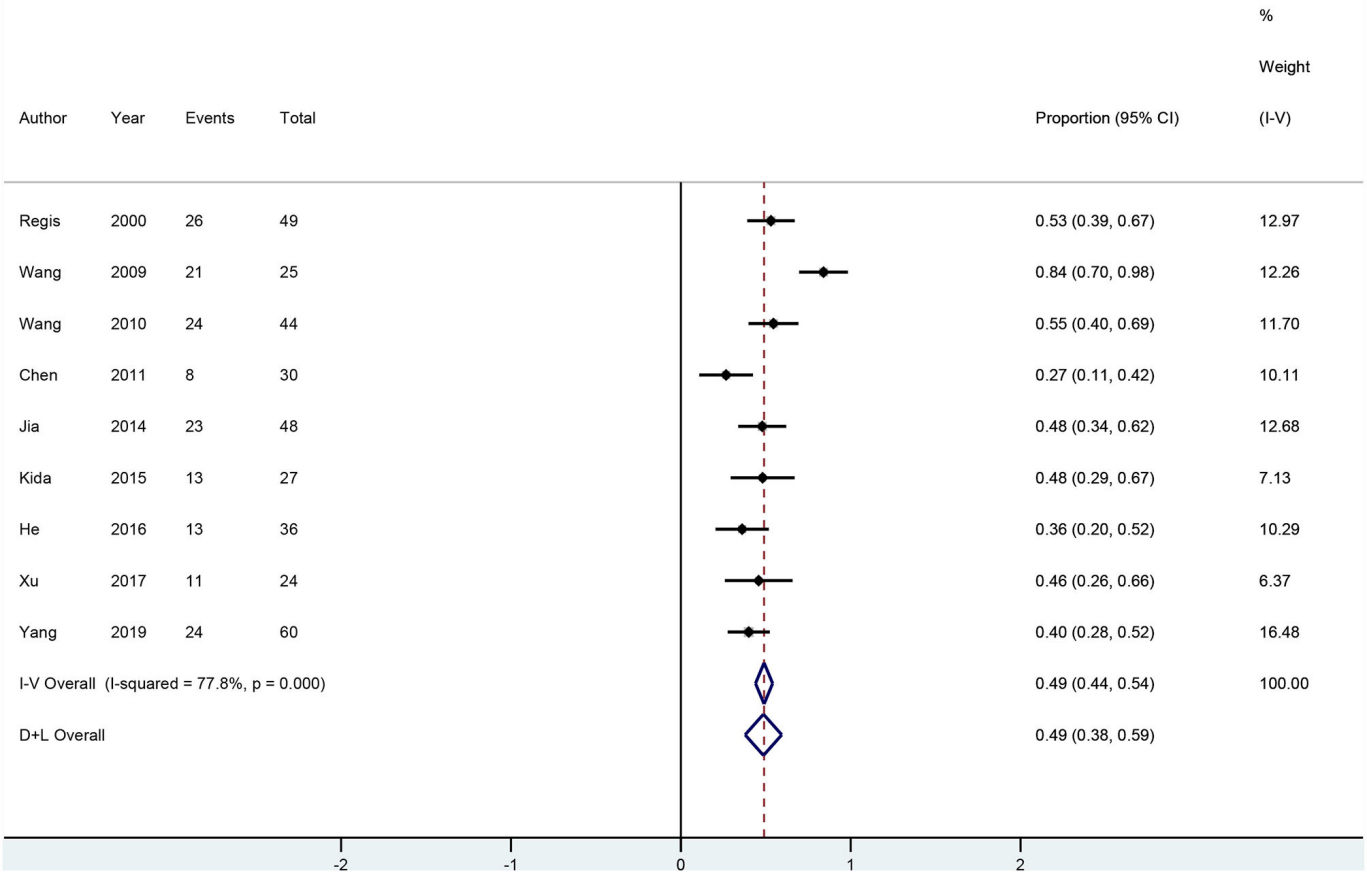
Forest plot of radiosurgery studies.

All 28 neurosurgery studies (except Baumann's study) showed that neurosurgery was an effective surgical treatment for seizures, with more than 50% of patients being classified as Engel class I. As shown in Figure 2, the overall proportion of patients in Engel class I was 0.79 (95% CI 0.75–0.83) across all 28 neurosurgery studies, which suggested that neurosurgery can significantly control seizures. Because *I*^2^ > 50%, we used a random effects model to analyze the data. The nine radiosurgery studies also demonstrated the efficacy of GKRS in the treatment of epileptogenic CCMs. As shown in Figure 3, the overall proportion of patients in Engel class I was 0.49 (95% CI 0.38–0.59). All radiosurgery studies were analyzed using a random effects model because *I*^2^ > 50%.

In addition, we performed subgroup analyses and the confounding factors in our studies were ethnicity, CCMs location and average lesion number (Table 3). Patients from North America (0.85, 95% CI 0.75–0.95), Asia (0.80, 95% CI 0.76–0.85) and Oceania (0.85, 95% CI 0.75–0.95) had higher proportions of favorable seizure outcomes in neurosurgery studies. When CCMs lesions were located in the frontal and temporal lobes, seizure outcomes of neurosurgery (0.78, 95% CI 0.40–0.99; 0.74, 95% CI 0.66–0.83; respectively) were significantly better than those of radiosurgery (0.56, 95% CI 0.39–0.73; 0.39, 95% CI 0.26–0.52; respectively). The effect of neurosurgery on single lesion (0.79, 95% CI 0.75–0.84) is better than that on multiple lesions (0.73, 95% CI 0.64–0.83). In contrast, the effect of neurosurgery on multiple lesions (0.47, 95% CI 0.36–0.59) is better than that on single lesion (0.35, 95% CI 0.27–0.44).

**Table 3:** Subgroup analysis.

	**Neurosurgery**		**Radiosurgery**	
**Subgroup**	**Number of cohorts**	**Proportion of patients in Engel class I (95%CI)**	**Number of cohorts**	**Proportion of patients in Engel class I (95%CI)**
**Ethnicity**				
European	7	0.68 (0.54–0.82)	1	0.53 (0.39–0.67)
North American	4	0.85 (0.75–0.95)	-	-
South American	1	0.62 (0.41–0.83)	-	-
Asian	15	0.80 (0.76–0.85)	8	0.48 (0.36–0.60)
Oceanian	1	0.85 (0.75–0.95)	-	-
**CCMs location**				
Frontal	4	0.78 (0.40–0.99)	3	0.56 (0.39–0.73)
Temporal	10	0.74 (0.66–0.83)	3	0.39 (0.26–0.52)
Parietal	4	0.62 (0.20–0.95)	3	0.52 (0.37–0.67)
Occipital	2	0.73 (0.00–1.00)	3	0.72 (0.23–0.99)
Others	1	1.00 (0.31–1.00)	2	0.85 (0.16–1.00)
**Average lesion number**				
1	4	0.79 (0.75–0.84)	3	0.35 (0.27–0.44)
>1	11	0.73 (0.64–0.83)	2	0.47 (0.36–0.59)

### Mortality and Morbidity

Of the 37 neurosurgery studies, thirty-three (89%) studies reported on the mortality or morbidity. Two (0.1%) patients died post-operatively, 212 (13.1%) patients experienced temporary morbidity, and 60 (4.4%) patients experienced permanent neurological symptoms. Eight (88.9%) of the nine radiosurgery studies reported on the mortality or morbidity. No deaths or permanent complications occurred. Thirty (17.1%) patients experienced temporary morbidity.

### Sensitivity Analysis

**Figure 4:**
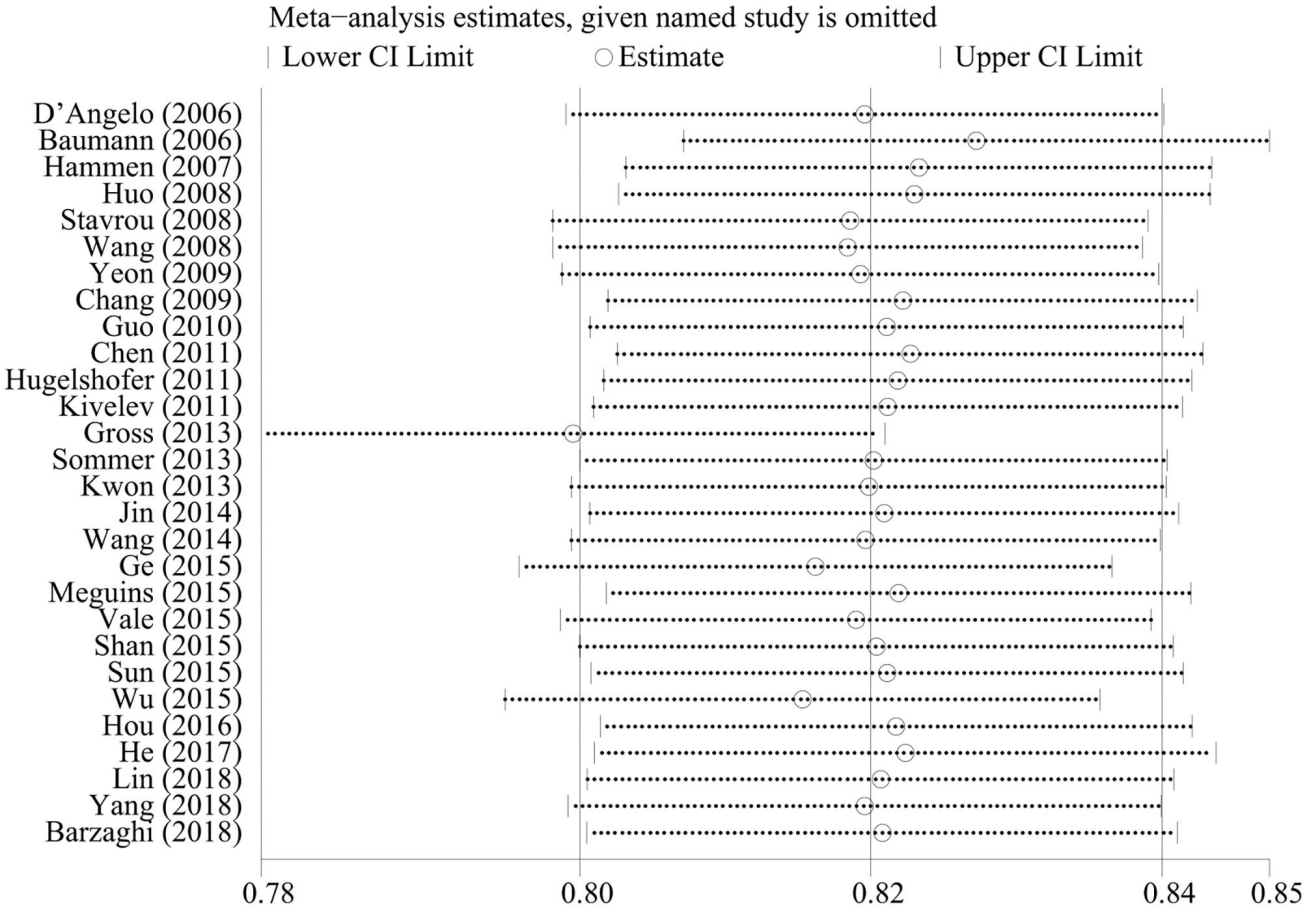
Sensitivity analysis of neurosurgery studies.

**Figure 5:**
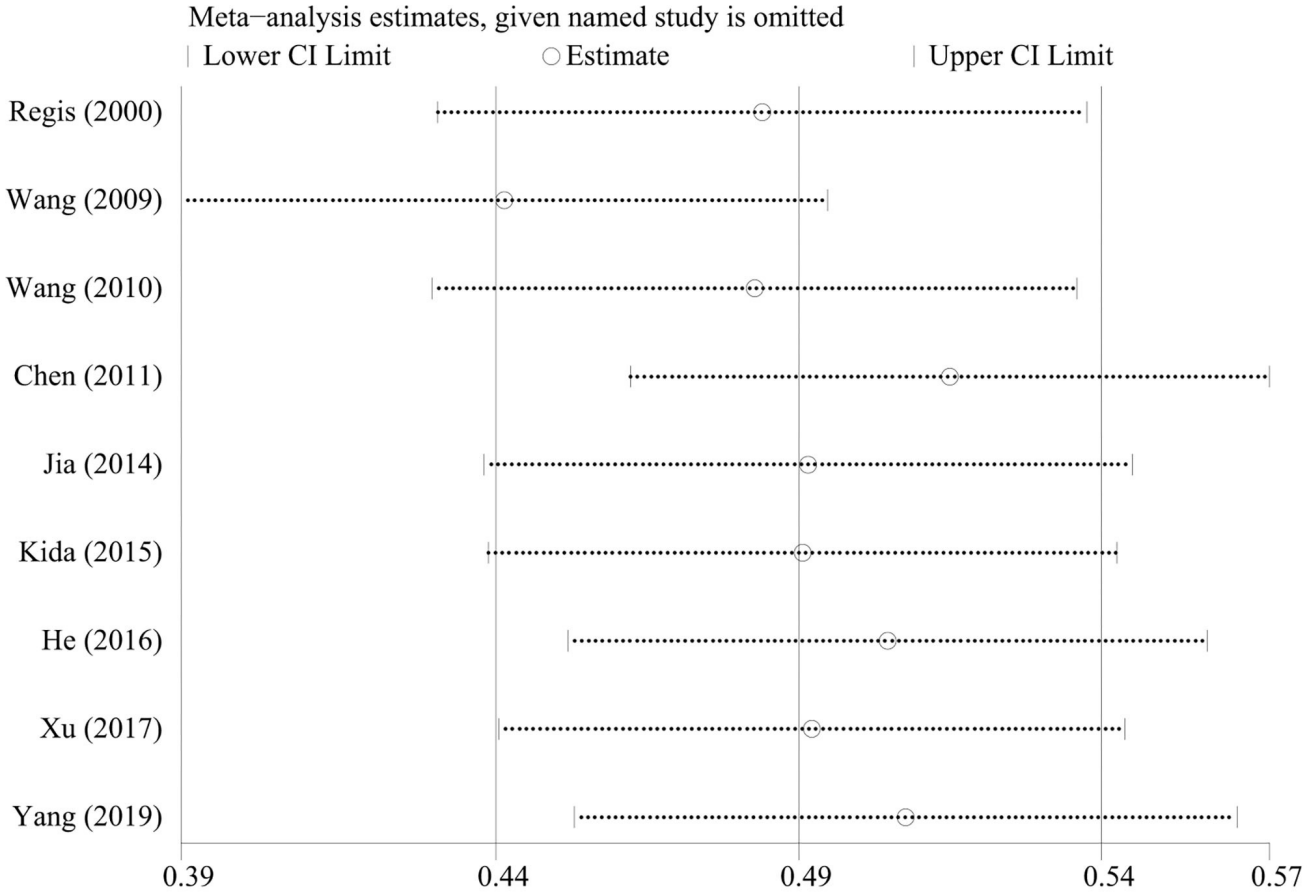
Sensitivity analysis of radiosurgery studies.

We omitted one study at a time to investigate the influence of a single study on the pooled estimates. The comparison results in the radiosurgery group were not significantly altered, indicating that this group's results were statistically robust. In the neurosurgery group, Gross's study was shown to have a substantial influence on the pooled estimates due to its higher proportion of patients in Engel class I. However, Gross's study did not affect our conclusions (Figures 4, 5).

### Publication Bias

**Figure 6:**
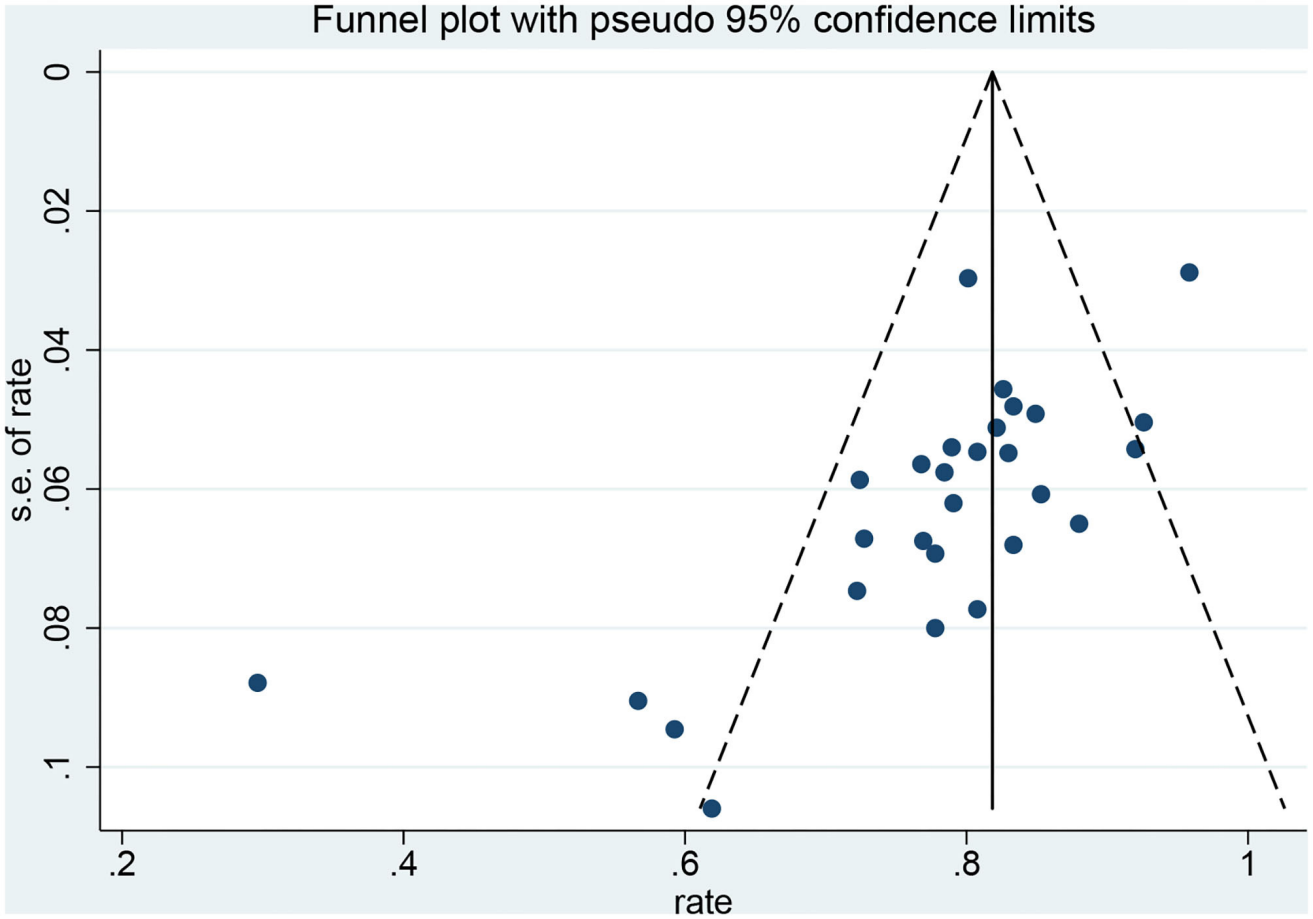
Funnel plot of neurosurgery studies.

**Figure 7:**
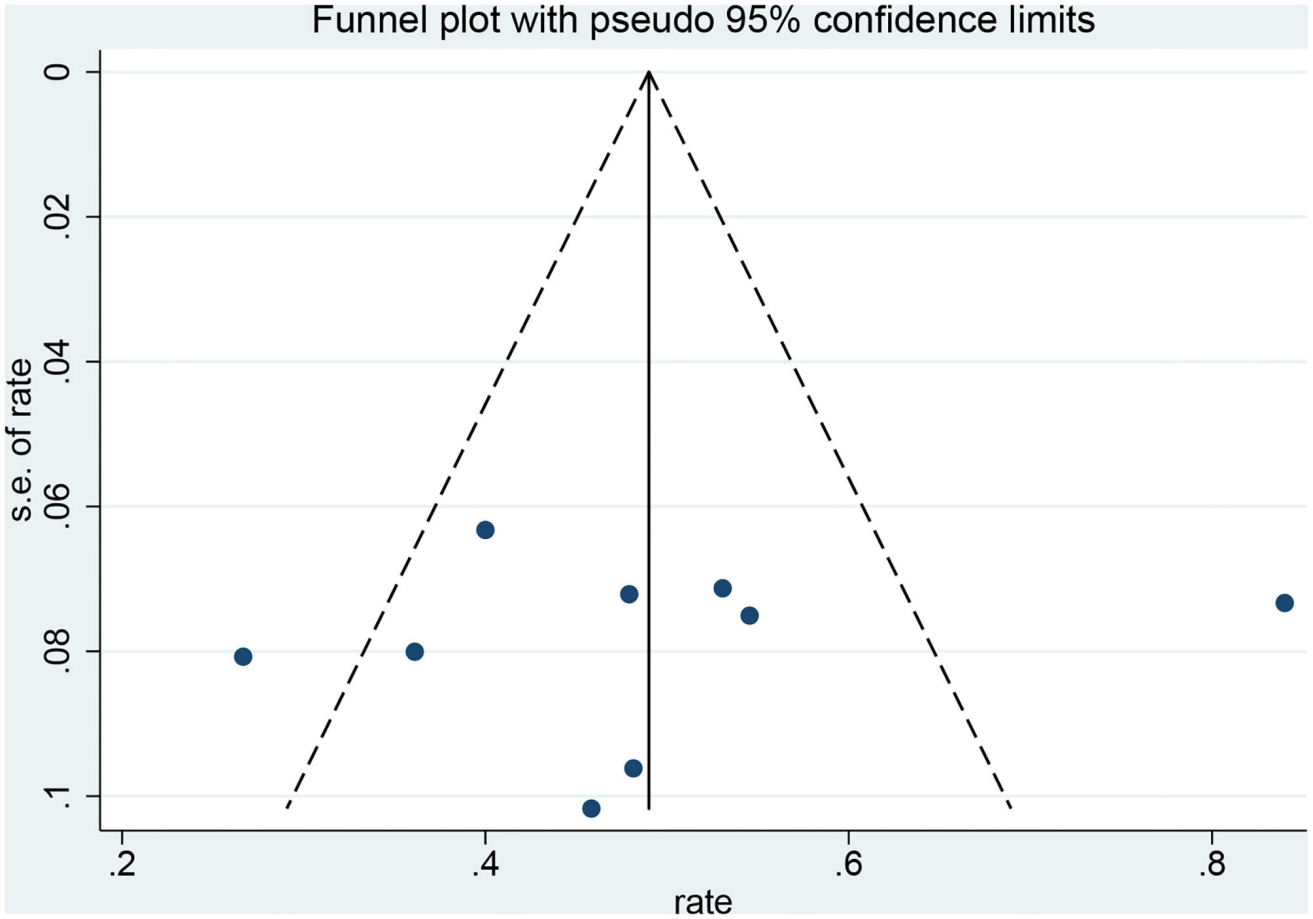
Funnel plot of radiosurgery studies.

Funnel plots and Egger's test were used to evaluate the publication bias. The *p*-values produced by Egger's test on the post-radiosurgery seizure outcomes and post-neurosurgery seizure outcomes were 0.778 and 0.000, respectively. Therefore, there was no publication bias in the radiosurgery studies, but publication bias might have influenced the results of the neurosurgery studies (Figures 6,7).

## Discussion

Overall, our results indicate that the epilepsy control rate after neurosurgery was higher than that after radiosurgery, but neurosurgery also had a relatively higher rate of permanent morbidity. The effect of neurosurgery on multiple lesions is better than that on single lesion whereas radiotherapy was the opposite. The effect of neurosurgery on frontal lobe and temporal lobe lesions is significantly better than those of radiotherapy. Ethnicity affects the seizure outcome following the treatment. Radiosurgery is more suitable for CCM lesions located in the parietal lobe and occipital lobe, while neurosurgery is more suitable for temporal lobe lesions.

CCMs are low-flow vascular malformations that are usually static and can also bleed repeatedly and grow. CCMs are occult vascular malformations that are difficult to find on DSA. MRI has a high specificity and sensitivity for CCMs, which can be clearly diagnosed and characterized due to their nodular or circular appearance. There is generally no edema or placeholder effect around the lesion except when it is accompanied by bleeding (69). The mechanism of CCM-induced epilepsy is still not fully understood. CCMs do not contain nerve tissues and will not become the epilepsy initiation area by itself. Peripheral hemosiderin deposition and gliosis caused by recurrent microhemorrhage of malformed vessels are considered to be the main causes of epilepsy (70).

AEDs are the primary treatment for CCMs with epilepsy. For refractory epilepsy, neurosurgery or radiosurgery should be considered. Yang's research shows that surgery for intractable epilepsy can effectively control seizures. In addition, the appropriate operation scheme can be selected according to the location of CCMs and the responsiveness of patients to antiepileptic drugs to maximize the control of epilepsy and minimize post-operative neurological sequelae (68). He et al. also reported the effectiveness of neurosurgery for intractable epilepsy and pointed out that the shorter the duration of seizures before surgery, the better the control of seizures after surgery (67). Ruan et al. (14) conducted a meta-analysis and the result showed that patients who underwent surrounding hemosiderin excision could exhibit significantly improved seizure outcomes compared to patients without hemosiderin excision. Additionally, Shang-Guan's meta-analysis reported that extended lesionectomy does not contribute to better seizure control for patients with cerebral cavernous malformations with epilepsy (15). In addition, radiotherapy can also be used for the treatment of refractory epilepsy. There has been a considerable amount of research on its effectiveness. Regis et al. showed that GKRS can control seizures safely and effectively. When CCMs are located in a highly functional area, the risk of surgical treatment is higher, and GKRS treatment is more appropriate (24). However, the ideal treatment remains unclear.

To compare the efficacy and safety of neurosurgery and radiosurgery for epileptogenic CCMs, we conducted a systematic review and meta-analysis of available data from published literature. The results of our systematic review showed that neurosurgery is more likely to be used in refractory epilepsy patients with CCM lesions located in the temporal lobe, while radiosurgery is more likely to be used in patients with CCM lesions located in the parietal lobe and occipital lobe. In addition, there was no significant difference in mortality and post-operative transient morbidity between the two treatments, but the proportion of patients with permanent complications was significantly higher in the neurosurgery group than in the radiosurgery group. Additionally, the results showed that 4.4% of patients in the neurosurgery studies experienced permanent morbidity, while no patients in the radiosurgery studies had permanent morbidity. We also found that the proportion of patients with temporary morbidity in the radiosurgery group (17.1%) was greater than that in the neurosurgery group (13.1%). After consulting the literature, we found that radiosurgery could cause to post-operative brain edema in patients, leading to a significantly higher proportion of patients suffering from temporary morbidity; however, brain edema will eventually subside over time.

The results of our meta-analysis showed that the seizure control rate was 0.79 (95% CI 0.75–0.83) for neurosurgery and 0.49 (95% CI 0.38–0.59) for radiosurgery. In terms of controlling epilepsy, the effect of neurosurgery is significantly better than that of radiosurgery. In addition, CCMs multiplicity and CCMs location are important factors affecting the prognosis of CCMs. Englot et al. (71) had reported that individuals with a single lesion received neurosurgery were more likely to attain post-operative seizure freedom. Some of the neurosurgery studies found CCMs locations were not related to seizure outcomes (38, 71). Wang et al. believed that radiosurgery is more effective for seizure caused by CCMs in frontal and parietal lobe than that caused in temporal lobe (40). Therefore, we performed subgroup analyses to summarize the influence of these confounding factors on the results. We observed that the effect of neurosurgery on single lesion (0.79, 95% CI 0.75–0.84) is better than that on multiple lesions (0.73, 95% CI 0.64–0.83), which further supported the conclusions of Englot et al. (71). On the contrary, we found that the effect of radiosurgery on multiple lesions (0.47, 95% CI 0.36–0.59) is better than that on single lesion (0.35, 95% CI 0.27–0.44). These data revealed that average lesion number is likely a factor influencing seizure outcome which needs further case-control trials. Consistent with previous studies, our results showed that there is little difference in the effect of neurosurgery on each site and radiotherapy was more effective for frontal (0.56, 95% CI 0.39–0.73) and parietal (0.52, 95% CI 0.37–0.67) CCMs than for temporal (0.39, 95% CI 0.26–0.52) CCMs. we also found that for lesions located in the frontal lobe and temporal lobe, neurosurgery (0.78, 95% CI 0.40–0.99; 0.74, 95% CI 0.66–0.83; respectively) is significantly superior to radiosurgery (0.56, 95% CI 0.39–0.73; 0.39, 95% CI 0.26–0.52; respectively). For CCMs lesions at other locations, the differences in seizure outcome between the two treatments were not significant.

The difference of gene background in CCMs patients is closely related to clinical manifestation and prognosis. Different ethnic groups have different genetic backgrounds and different mutation sites (72). Previous cohort studies have not focused on this. Therefore, we did a subgroup analysis and our data indicated that North Americans (0.85, 95% CI 0.75–0.95), Asians (0.80, 95% CI 0.76–0.85) and Oceanians (0.85, 95% CI 0.75–0.95) benefited more from neurosurgery than Europeans (0.68, 95% CI 0.54–0.82) and South Americans (0.62, 95% CI 0.41–0.83). We speculated that ethnicity might be associated with prognosis and further random controlled trails were needed. Unfortunately, data on mortality and morbidity of the two treatment could not be subgroup analyzed as they were not provided in the majority of the included studies.

Lately, there is an emerging minimally invasive technique called stereotactic laser ablation (SLA) which is getting into focus. SLA could precisely ablate lesions with less collateral injury around lesions. A cohort study by Willie et al. (73) reported 17 patients receiving SLA, 14 (82%) of whom achieved Engel I after a year-long follow-up period. SLA has the same good seizure control rate as neurosurgery and is more tolerable for the patients. Therefore, SLA is expected to be a first-line minimally invasive therapy for CCMs-related epilepsy, but more case-control trials are still needed.

The NOS was used to assess the quality of the included studies, and each study had a moderate level of quality with an average score of 6. Our systematic review and meta-analysis has three limitations. First, all the included studies were retrospective studies. Therefore, randomized controlled trials are urgently needed. Second, neurosurgery was not consistent in all the included studies. Last, the experience of surgeons greatly affects the outcome of the operation.

## Conclusion

In summary, our paper demonstrates that the epilepsy control rate after neurosurgery was higher than that after radiosurgery, but neurosurgery also had a relatively higher rate of permanent morbidity. Number of lesions, location and ethnicity are likely significant factors influencing the seizure outcome following treatment. Therefore, our data provide new ideas for clinical individualized precision medicine but further random controlled trials are still needed.

## Data Availability Statement

All datasets presented in this study are included in the article/Supplementary Material.

## Author Contributions

XG, KY, and JS contributed conception and design of the study. PL and XJ organized the database. YC performed the statistical analysis. XG wrote the first draft of the manuscript. BZ, HZ, SD, LZ, and PL wrote sections of the manuscript. All authors contributed to manuscript revision, read, and approved the submitted version.

## Conflict of Interest

The authors declare that the research was conducted in the absence of any commercial or financial relationships that could be construed as a potential conflict of interest.
